# Clinical Significance of Preterm Singleton Pregnancies Complicated by Placental Abruption following Preterm Premature Rupture of Membranes Compared with Those without p-PROM

**DOI:** 10.5402/2012/856971

**Published:** 2012-05-28

**Authors:** Shunji Suzuki

**Affiliations:** Department of Obstetrics and Gynecology, Japanese Red Cross Katsushika Maternity Hospital, 5-11-12 Tateishi, Katsushika-ku, Tokyo 124-0012, Japan

## Abstract

The purpose of this paper was to examine the obstetric and neonatal outcomes of preterm singleton pregnancies complicated by placental abruption following preterm premature rupture of membranes (p-PROM) compared with those without p-PROM. We reviewed the obstetric records of 95 singleton deliveries complicated by placental abruption at 22–36 weeks' gestation. The incidence of placental abruption in singleton pregnancies with p-PROM was 4.7%, and the crude odds ratio of placental abruption for women following p-PROM was 6.50 (*P* < 0.01). Of the 95 cases of placental abruption in preterm singleton deliveries, 64 cases (67.4%) occurred without p-PROM and 31 cases (32.6%) occurred following p-PROM. The incidence of histological chorioamnionitis stage III in the patients following p-PROM was significantly higher than that in the patients without p-PROM (*P* = 0.02). The rate of emergency Cesarean deliveries associated with nonreassuring fetal status (NRFS) in the patients following p-PROM was significantly lower than that in the patients without p-PROM. However, there were no significant differences in the maternal and neonatal outcomes between the patients with and without p-PROM. Although p-PROM may be one of important risk factors for placental abruption associated with chorioamnionitis, it may not influence the perinatal outcomes in preterm placental abruption.

## 1. Introduction

Placental abruption or premature separation of the normally implanted placenta is a serious and life-threatening obstetric complication for both mother and fetus [1–3]. Although the cause of placental abruption remains elusive, the presence of inflammation and infection has been suggested to be the primary cause of placental abruption [2, 4–8]. Some previous studies have observed an association between intrauterine infection, especially chorioamnionitis (CAM), and placental abruption [2, 4–8]. In addition, evidence from prior studies suggests that women exposed to prolonged preterm premature rupture of membranes (p-PROM) are at increased risk of placental abruption [9–11], because recent evidence has linked neutrophil infiltration into the deciduas with preterm placental abruption [2, 7]. In our earlier studies [12, 13], for example, the incidence of preterm delivery, p-PROM, and low birth weight in the cases of placental abruption with chorioamnionitis were higher than in cases without chorioamnionitis: however there were no significant differences in the incidence of other outcomes between the cases of placental abruption with and without histological chorioamnionitis. However, there have been few examinations concerning the influence of precedent p-PROM on the severity of placental abruption at preterm only.

In this study, we examined the obstetric and neonatal outcomes of preterm singleton pregnancies complicated by placental abruption following p-PROM compared with those without p-PROM.

## 2. Patients and Methods

The protocol for this paper was approved by the Ethics Committee of the Japanese Red Cross Katsushika Maternity Hospital. In addition, informed consent concerning analysis from a retrospective database was obtained from each subject at their first hospital visit.

All subjects in this study had received care at Japanese Red Cross Katsushika Maternity Hospital between April 2002 and March 2011. We reviewed the obstetric records of 95 singleton deliveries complicated by placental abruption, defined as complete or partial separation of a normally implanted placenta indicated by evidence of retro-placental bleeding at 22–36 weeks' gestation. (In our hospital, there were 65 cases complicated by placental abruption after 37 weeks' gestation during the 9-year period.) We excluded the cases referred from other hospitals after the onset of placental abruption. In this paper, we examined the incidence of hypertensive disorders such as gestational hypertension and preeclampsia, emergency Cesarean delivery, disseminated intravascular coagulation (DIC), maternal blood loss requiring hemotransfusion, small-for-gestational-age infants, fetal demise, nonreassuring fetal status (NRFS), Apgar score <4 at 1 and 5 minutes and umbilical artery pH < 7. Infants who were small-for-gestational-age were defined as those with sex- and age-adjusted birth weights below the tenth percentile according to the neonatal birth weight standards for gestational age in Japanese [14]. In addition, microscopic histological analyses of the placentas were performed to diagnose chorioamnionitis (CAM). The severity of CAM, that is, inflammation of the placental surface, was determined by the degree of maternal polymorphonuclear lymphocyte infiltration into either the subchorionic space (intervillositis: stage I), the intervillous space (chorionitis: stage II) or the amniotic cavity (CAM in a narrow sense: stage III) according to Blanc's criteria [15]. 

### 2.1. Analysis

Data are presented as number (%) or mean ± SD. For statistical analysis, the *χ* ^2^ test with Yates' correction for categorical variables was used. While the Student's *t*-test for continuous variables was used. Odds ratios (ORs) and 95% confidence intervals (CIs) were also calculated. Differences with *P* < 0.05 were considered significant.

## 3. Results

Of the 95 cases of placental abruption in deliveries at 22–36 weeks' gestation, 64 cases (67.4%) occurred without p-PROM and 31 cases (32.6%) occurred following p-PROM.

During the 9-year period, there were 17,667 singleton deliveries after 22 weeks' gestation in our hospital. Of these, 655 cases were complicated by p-PROM (3.7%). In our hospital, therefore, the incidence of placental abruption in singleton pregnancies with p-PROM was 4.7% and the crude OR of placental abruption for women following p-PROM was 6.50 (95% CI: 4.4–9.7, *P* < 0.01).

Table 1 shows the perinatal outcomes of preterm singleton pregnancies complicated by placental abruption with and without p-PROM. The incidence of histological CAM stage III in the patients following p-PROM was significantly higher than that in the patients without p-PROM (crude OR: 5.22, 95% CI: 1.4–19, *P* = 0.02). On the other hand, the rate of emergency Cesarean deliveries due to NRFS in the patients following p-PROM was significantly lower than that in the patients without p-PROM (emergency Cesarean delivery, crude OR: 0.13, 95% CI: 0.05–0.33, *P* < 0.01; NRFS, crude OR: 0.34, 95% CI: 0.13–0.87, *P* = 0.04). However, there were no significant differences in the maternal and neonatal outcomes between the patients with and without p-PROM.

## 4. Discussion

The major findings of the current study are: (1) the preterm singleton pregnancies complicated by placental abruption following p-PROM was strongly associated with the presence of histological CAM more than those without p-PROM, and (2) the perinatal outcomes of preterm placental abruption following p-PROM were not different from those without p-PROM at preterm.

 Some previous studies have reported that p-PROM is a common obstetric complication, occurring in approximately 1-2% of pregnancies, and it is one of important risk factors for placental abruption [9–11]. Our current results also support these previous studies.

Histological CAM, defined as inflammation of the extraplacental membrane, has been consistently linked with prematurity and low birth weight of neonates [16]. In addition, the relationship between histological CAM and infection (positive culture) of the chorioamnion has been reported to be strongest among preterm deliveries [17, 18]. In cases with histological CAM, because the prematurely delivered placentas have been observed to be often accompanied by an acute marginal hemorrhage that undermines the edge of the placenta and that originates from deciduitis [2]. This hemorrhage process can cause premature labor and/or p-PROM and has been reported to differ from the typical placental abruption due to other causes such as preeclampsia [5]. Vintzileos et al. [8] also suggested that true placental abruption following the presence of CAM usually occurs after PROM. On the other hand, Nelson et al. [9] speculated that an acute reduction in the uterine volume and intrauterine surface as a consequence of p-PROM could ultimately lead to disruption of the site of placental attachment in the decidual spongiosa layer, thereby predisposing to abruption. In this study, unfortunately, we could not examine the intervals between p-PROM and onset of placental abruption. However, the progress of placental abruption in the cases following p-PROM may not tend to be acute; because the rate of cases with NRFS requiring cesarean operation in women following p-PROM was lower than those without p-PROM. Therefore, our results support the previous studies suggesting the association among CAM, p-PROM, and placental abruption [10, 11].

In this study, the perinatal outcomes of preterm placental abruption following p-PROM were not different from those without p-PROM at preterm, although the rate of emergency Cesarean delivery due to NRFS in cases following p-PROM was lower than in those without p-PROM. One reason may be the small sample size in this study. The other possible reason is that a more rapid Cesarean delivery might tend to be carried out in cases without PROM due to more typical symptoms, (bleeding, abdominal pain, and NRFS) and more easy diagnosis of placental abruption compared with cases following PROM [19]. Because rupture of membranes (amniotomy) has been supposed to decrease bleeding from the implantation site of the placenta and reduce entry of thromboplastin into the maternal circulation [19]. In our series, therefore, the influence of placental abruption on maternal and fetal conditions might be larger in cases without PROM than those following PROM; however, a rapid delivery of the fetus by Cesarean section might prevent serious complications in many cases without PROM.

In conclusion, although p-PROM may be one of important risk factors for placental abruption associated with CAM, it may not influence the perinatal outcomes in preterm placental abruption.

## Figures and Tables

**Table 1:** Obstetric complications and perinatal outcomes of preterm singleton pregnancies complicated by placental abruption with and without preterm premature rupture of membranes (p-PROM).

	p-PROM		*P* value*
	(−)	(+)	
*N*	60	42	
Maternal age (years)	31.9 ± 4.4	30.5 ± 4.6	0.16
Parity	0.8 ± 1.1	0.7 ± 0.7	0.59
Multiparous	32 (50.0)	18 (58.1)	0.61
*Gestational age at delivery*			
Average (weeks)	33.3 ± 2.6	32.5 ± 3.5	0.26
<32 weeks	12 (18.8)	10 (32.3)	0.23
Hypertensive disorders	6 (9.4)	0 (0.0)	0.19
Emergency Cesarean delivery	52 (81.3)	11 (35.5)	<0.01
DIC	12 (18.8)	1 (3.2)	0.08
Blood loss requiring transfusion	13 (20.3)	3 (9.7)	0.34
Histological chorioamnionitis**			
Stage II	14 (21.9)	9 (29.0)	0.61
Stage III	4 (6.3)	8 (25.8)	0.02
*Neonatal birth weight*			
Average (g)	2,006 ± 544	1,891 ± 642	0.39
Small for gestational age	11 (17.2)	6 (19.4)	0.98
Fetal demise	9 (14.1)	1 (3.2)	0.21
*Surviving fetuses/neonates*			
*n*	55 (85.9)	30 (96.8)	0.21
NRFS	41 (74.5)	15 (50.0)	0.04
Apgar 1′ < 4	14 (25.5)	2 (6.7)	0.07
Apgar 5′ < 4	4 (7.3)	1 (3.3)	0.77
Umbilical artery pH < 7	9 (16.4)	1 (3.3)	0.14

Values are expressed as *n* (%) or mean ± SD.

*P values by Student's *t*-test or *χ* ^2^ test.

**Microscopic histological analysis of the placenta.

p-PROM: preterm premature rupture of membranes.

DIC: disseminated intravascular coagulation.

NRFS: nonreassuring fetal status.
